# The Effect of New Cooperative Medical Scheme on Health Outcomes and Alleviating Catastrophic Health Expenditure in China: A Systematic Review

**DOI:** 10.1371/journal.pone.0040850

**Published:** 2012-08-20

**Authors:** Xiaoyun Liang, Hong Guo, Chenggang Jin, Xiaoxia Peng, Xiulan Zhang

**Affiliations:** 1 School of Social Development and Public Policy, Beijing Normal University, Beijing, China; 2 School of Public Health and Family Medicine, Capital Medical University, Beijing, China; Tehran University of Medical Sciences, Islamic Republic of Iran

## Abstract

**Background:**

In 2002, the Chinese government launched a new rural health financing policy to provide health insurance (New Cooperative Medical Scheme, NCMS) for its rural population. NCMS, jointly financed by governments and individual households, aims to protect households from impoverishment due to catastrophic health expenditure. In 2011, NCMS covered more than 96% of the rural population. We have systematically searched and reviewed available evidence to estimate the effects of NCMS on health outcomes and on alleviating catastrophic health expenditure.

**Methods:**

PubMed, Web of Science with Conference Proceedings, ProQuest Digital Dissertations, CMCI, CNKI, and VIP were searched. We also obtained literature from colleague communications. Quasi-experimental studies regarding the effect of NCMS on health outcomes and catastrophic health expenditure were included. Two independent reviewers screened the literature, extracted the data, and assessed the study quality.

**Results:**

Fifteen studies out of the 6123 studies in the literature fulfilled criteria and were included in this review. Twelve studies identified the relationship between NCMS and health outcomes, among which six studies measured sickness or injury in the past four weeks, four measured sickness or injury in the past two weeks, and five measured self-reported health status. Four studies focused on the relationship between NCMS and alleviating catastrophic health expenditure. However, the results from these studies were in conflict: individual studies indicated that NCMS had positive, negative, or no effect on health outcomes and/or the incidence of catastrophic health payments, respectively.

**Conclusions:**

We still have no clear evidence that NCMS improves the health outcomes and decreases the alleviating catastrophic health expenditure of the China’s rural population. In addition, the heterogeneity among individual studies reminds us that provider payment method reforms, benefit package and information systems around NCMS should be improved in the future.

## Introduction

### 1. Background of New Cooperative Medical Scheme (NCMS)

Under China’s planned economy, almost all rural residents were covered by the Cooperative Medical Scheme (CMS). Moreover it was believed that the program helped to reduce China’s mortality rate during the 1960s and 1970s [1]. In the 1980s, China started its transition from a planned to a market economy. The proportion of the rural population with CMS coverage dramatically declined. By 1993 only 6.6% of the rural residents had CMS coverage [2].

In 2002, the Chinese government launched a new rural health financing policy to provide health insurance (NCMS) for its rural population. NCMS is financed by the combined contributions from central and local governments, as well as from individual households. NCMS aims to protect households from becoming impoverished due to catastrophic health expenditure [3]. Inpatient care was covered from the beginning of NCMS. General outpatient expenses as well as large outpatient expenses due to selected chronic diseases have been reimbursed from the pooling revenue in recent years. In 2009, NCMS reimbursed for general outpatient expenses in 41% of NCMS counties, while two-thirds of counties covered catastrophic outpatient costs [4]. In 2011, NCMS covered 832 million people, more than 96% of China’s rural population [5]. The strong support for universal NCMS from governments reflects the assumption that lack of insurance is responsible for poor health, which may in turn lead to catastrophic financial payments and subsequent impoverishment. However, there is insufficient evidence to confirm the effect of NCMS on improving health outcome and on alleviating catastrophic health expenditure.

One concern with NCMS is that its financing is too small to make a significant impact on either health outcomes and/or reducing catastrophic health expenditure. At its inception, the minimum financial requirement is 50 RMB (1 USD ≈ 8.26 RMB, Jan.2003) per person. In 2009, the average financial requirement per person reached 113.37 RMB(1 USD ≈ 6.81 RMB, Jan.2009) [6]. As a result of limited financing, many services are not, or only partially covered, deductibles are high, ceilings are low, and coinsurance rates are high. In addition, because NCMS encourages people to seek medical care, providers may over-prescribe drugs and high-tech care due to its fee-for-service payment system and the limited financing for the health facilities from the government [7]. NCMS coverage may not result in reducing out-of-pocket expenditures. We now turn to the evidence of the impact of NCMS on health outcomes and catastrophic health expenditures.

### 2. NCMS and Health Outcome

Most of the studies show that the impact of NCMS is limited [8], [9], [10]. Lei and Lin used a longitudinal sample drawn from the China Health and Nutrition Survey (CHNS) and found that NCMS did not improve the health status of enrollees, as measured by self-reported health status and by sickness or injury in the past four weeks [8]. Another study indicated that disease prevalence in the past two weeks increased from 63.0‰ to 86.9‰ after introducing NCMS [9]. Yuan et al. found similar results [10].

### 3. NCMS and Catastrophic Health Expenditure

Although the main objective of NCMS is to prevent rural residents from being impoverished by medical expenses, the 2003 to 2005 longitudinal study by the Chinese Ministry of Health and the World Bank indicated that inpatient cost per case increased by 30% after people were insured [11]. Wagstaff et al. similarly found that NCMS increased the incidence of catastrophic household out-of-pocket payments [12]. In addition, provincial studies showed that farmers’ average annual inpatient expenses in Jiangxi province (located in middle China) increased quickly, by 26.2% from the year 2004 to 2008, and the highest proportion of claims was 30.94% in 2008. It appears that most of the reimbursement from the NCMS was offset by the growth of medical expenses [13]. Other studies showed that NCMS reduced impoverishment due to healthcare payment [14], [15]. Sun et al. indicated that the poverty incidence due to catastrophic medical payment was reduced by 19.81% after NCMS was introduced in Shandong province [15].

A literature of nearly 5000 items cover the effect of NCMS on health outcomes and/or catastrophic heal expenditure between the national pilot in 2003 to the end of 2010. There is, however, no systematic review on this topic. Many studies used counterfactual analysis to compare catastrophic payments before and after NCMS reimbursements. One cannot draw causal inference about the effect of the NCMS on health outcomes/catastrophic health expenditure [16], [17] from these studies. Some reviews have addressed associations between insurance and health outcome and/or reducing catastrophic health expenditure [18]. However, the evidence from these reviews is hampered by heterogeneous study designs. So comprehensive understanding of evidence is needed for further studies and policy making [19].

### 4. Objectives

The objective of a systematic review was to determine the impact of New Cooperative Medical Scheme (NCMS) on health outcomes and/or catastrophic health expenditure in China.

## Methods

### 1. Criteria for Considering Studies for Systematic Review

- Types of studies

Quantitative studies, mainly quasi-experiment studies, which estimate the causal effects of NCMS on health outcomes and on reducing catastrophic health expenditure in China. In the absence of findings from randomized controlled trials, well designed and implemented quasi-experimental studies could estimate causal effects of health insurance [20]. The design of quasi-experimental studies included posttest-only design with control group, repeated cross-sectional study, and pre-post design with control group.

- Types of participants

Rural residents.

- Types of intervention

NCMS.

- Comparison

NCMS vs. non-NCMS. The control group included internal control (the subjects were in the NCMS implemented county but did not enrolled in the NCMS), and external control (the subjects were not in the NCMS implemented county).

- Types of outcomes measurement

1. Health outcomes: prevalence of sickness or injury in the last two or four weeks, and the proportion of self-reported health, and mortality;
2. Catastrophic health expenditure and its threshold (10%, 20%, 30%, 40%, 50%, 60% of household capacity to pay, CTP)
3. Catastrophic health expenditure: Payment is considered catastrophic when a household has to cut its basic living expenses over one year in order to afford the medical expenses of its household member(s) [21].

- Languages

Publications in both Chinese and English were included.

- Exclusion criteria

1. Because we are interested in whether NCMS improves health outcomes and/or reduces the household catastrophic health expenditure, we exclude literature on NCMS regulation, financing, supervision of the NCMS fund, capacity of healthcare facilities, participation proportions, and establishing impact or performance evaluation indicators. In addition, regarding the effect of NCMS, we also exclude health service utilization, patient’s satisfaction with NCMS, inpatient and outpatient reimbursement proportions, determinants of participation and sustainability, and the insured’s benefit proportion.
2. This review excluded the studies without individual or household data.
3. This review excluded the studies that: (1) used prevalence of chronic disease as indicators of health outcomes; (2) used medical care financed by borrowing or selling assets as indicators of catastrophic health expenditure; (3) used self-comparison before and after reimbursement by NCMS as indicators of catastrophic health expenditure; (4) compared NCMS and other health insurance on health outcomes and catastrophic health expenditure. These studies commonly evaluated the effect of NCMS on health outcomes and reduced catastrophic health expenditure.

### 2. Search Strategy

We searched the following databases: CNKI (China National Knowledge Infrastructure); Chongqing VIP database (a full text issues database of China), CMCI on Chinese Biomedical Literature; PubMed; ISI Web of Science with Conference Proceedings; ProQuest Dissertations and thesis. Books and working papers were obtained from communication with colleagues. We included Chinese and English literature published between January 2003 and December 2010. The search combined the following two categories of terms:

- The terms describing NCMS: (a) New Cooperative Medical Scheme; (b) New Cooperative Medical System; (c) New Rural Cooperative Medical Scheme; (d) New Rural Cooperative Medical System.
- The terms describing outcome: (a) health; (b) cost; (c) expenditure; (d) catastrophic.

Our detailed search strategy is in Appendix S1.

### 3. Screening and Selection of Literature

Primary, selected studies were based on the inclusion criteria described above. An EndNote database containing the search results was used to keep track of references identified through the electronic database search (to be screened for inclusion).

Screening of literature proceeded at two levels. At level 1 screening, two reviewers (Hong Guo and Xiaoyun Liang) independently performed an assessment of the identified records by reading the title/abstract. The pre-developed inclusion questions for level 1 were based on the inclusion criteria listed in Appendix S2. Records not excluded at level 1 were promoted to level 2 screening. Differences in opinion at level 1 screening were resolved by promoting the record to level 2 screening.

At level 2 screening, two reviewers (Hong Guo and Xiaoyun Liang) independently evaluated the full text of each record promoted from level 1 screening for inclusion. They used a pre-developed inclusion form (Appendix S3). Differences in assessment at level 2 screening were discussed until consensus was reached. If consensus could not be reached, then a consulting group, including two experts (Xiaoxia Peng and Chenggang Jin), was asked to resolve disagreements. If the reviewers score “Unclear” to any one question, the inclusion question was resolved by re-reading of the text, discussion and consensus (or resolved by a third person if consensus could not be reached). The main reason for exclusion at this stage was recorded for each record, and a list of excluded records (with reasons) was created.

When there was more than one record of the same study, we included all potential eligible records according to the inclusion criteria, but used the most relevant one as the main record.

### 4. Data Extraction

Two reviewers independently extracted data from the published sources using the pre-designed data extraction form.

After independent extraction of data, two reviewers compared their forms, and resolved any discrepancies by the consulting experts.

### 5. Quality Assessment

Two reviewers independently assessed the quality of the selected studies using quality assessment criteria for quasi-experiment study adapted from Loevinsohn (1990) [22], Thomas et al. (2004) [23], and Gersten et al. (2005) [24] (Appendix S4). Next, they compared and discussed their assessments. If consensus could not be reached, consulting experts (Xiaoxia Peng and Chenggang Jin) were asked to resolve disagreements. After discussion, a final decision of yes, no or unclear of methodological quality was agreed upon by the reviewers.

### 6. Literature Synthesis

From each included study, we abstracted information about author, year data were collected, study design, sample site and data source, sample size, measures of the type of dependant variable (health status/catastrophic health expenditure), statistical analysis, and findings relevant to the effects of NCMS on health outcomes/catastrophic health expenditure. The primary comparison of the effects of insurance was explored: differences between rural population with and without NCMS, and control groups included internal controls and external controls. Substantial variation of methods among individual studies precluded formal meta-analyses. Therefore, results are presented in narrative form.

## Results

**Figure 1:**
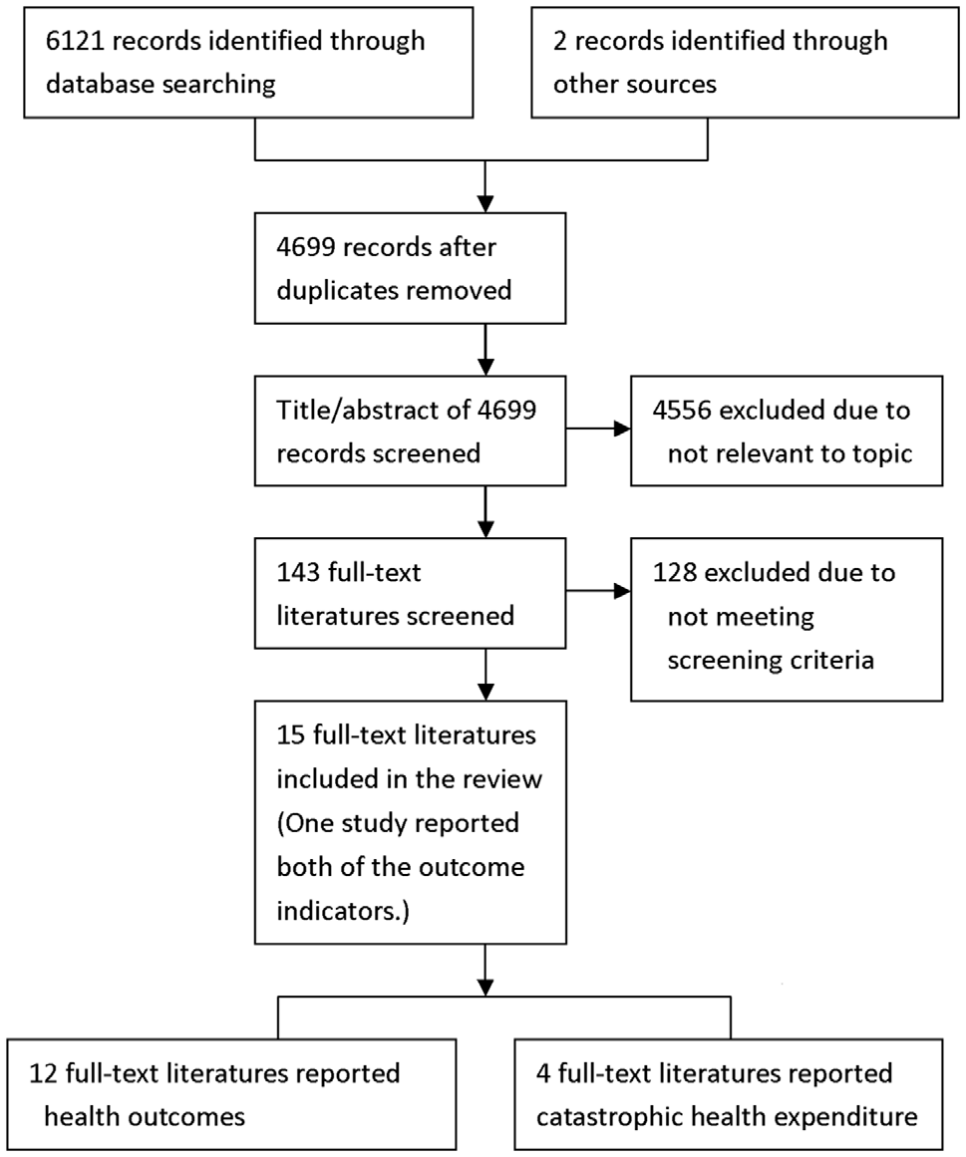
Flow diagram for selection of studies.

In total, 6123 studies were identified: 4107 from CNKI; 1329 from Chongqing VIP database; 598 from CMCI; 24 from PubMed; 57 from ISI; 6 from ProQuest; and 2 from colleague communication (Table S1). There were 4699 studies after duplicates removed, and 4556 were excluded due to not being relevant to topic. Among the 143 potential eligible studies, 15 studies [8], [25], [26], [27], [28], [29], [30], [31], [32], [33], [34], [35], [36], [37], [38] fulfilled level 1 and level 2 screening (Figure 1).

### 1. Characteristics of the Included Studies

**Figure 2:**
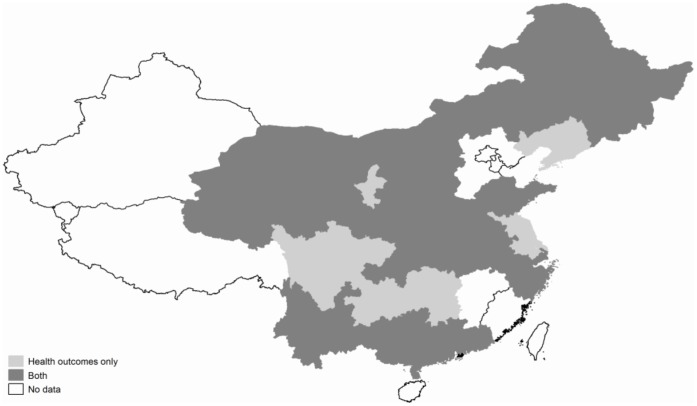
Study site of the 15 studies.

Of the included fifteen studies, twelve studies reported health outcomes [8], [25], [26], [27], [28], [29], [30], [31], [32], [33], [36], [38], and four reported catastrophic health expenditure [33], [34], [35], [37] (One study measured both outcome indicators [33]). Six studies used posttest-only design with control group [26], [27], [31], [32], [34], [38], six used repeated cross-sectional design [8], [28], [29], [33], [35], [37], and three used pre-post design with control group [25], [30], [36]. Five studies utilized data from China Health and Nutrition Survey (CHNS) [8], [25], [28], [30], [36]. All of the included studies were published from 2005 to 2010, and the year that data were collected is from 2003 to 2007. Figure 2 shows the geographical distribution of the fifteen included studies, which covered most of the provinces in the mainland of China. Eight provinces were not covered: Tibet, Xinjiang, Fujian, Jiangxi, Beijing, Tianjin, Hebei and Hainan province.

### 2. Quality Assessment of the Included Studies

**Figure 3:**
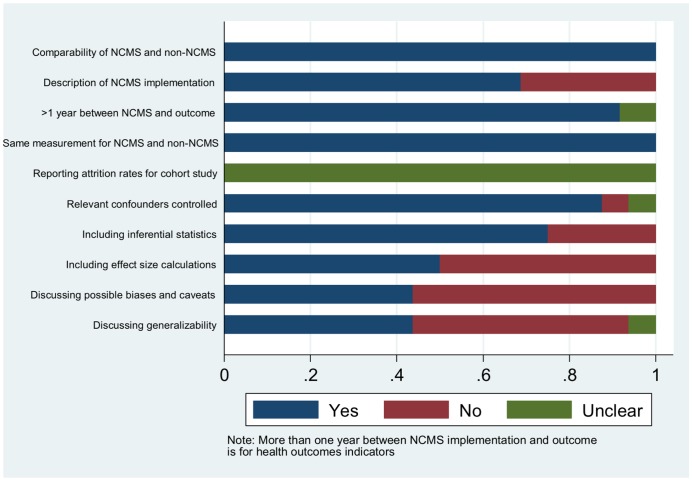
Quality assessment for included literatures.

As Figure 3 shows, all studies described the procedures used to increase the likelihood that relevant characteristics of participants in the sample were comparable between NCMS and non-NCMS members. For the twelve studies focused on health outcomes, all of the studies (apart from one [32]) showed that the period between the implementation of NCMS and health outcomes was more than one year. None of the studies using pre-post design with a control group reported their attrition rates. Fourteen studies (87.5%) utilized stratification, matching or analysis to control relevant confounding factors. For example, propensity score matching (PSM) with the double difference (DD) method was used to estimate the effect of NCMS [8], [35], [36]. 50% of the studies included inferential statistics and effect size calculations [8], [25], [26], [28], [32], [33], [35], [36]. Seven (43.75%) studies included a discussion of possible bias [8], [25], [28], [34], [35], [36], [38], and seven studies discussed the generalizability of the results [8], [25], [27], [28], [29], [35], [38].

### 3. NCMS and Health Outcomes

Of the twelve studies measuring health outcomes, six [8], [25], [28], [30], [32], [38] measured sickness or injury in the past four weeks, four [26], [27], [29], [33] measured sickness or injury in the past two weeks, five [8], [25], [31], [33], [36] measured self-reported health. None measured mortality (Table 1).

**Table 1:** Characteristics of quasi-experimental studies estimating effects of NCMS on health outcomes.

Author	Year data collected	Study design	Sample site and data source	sample size	Measures	Statistic analysis	Findings	Outcomes	Study quality
Lei & Lin (2009)[8]	2000, 2004, 2006	O×OO×ORepeated cross-sectional design	CHNS*Liaoning, Heilongjiang, Jiangsu, Shandong, Henan, Hubei, Hunan, Guangxi, and Guizhou province	17 984 individuals in 36 counties of 9 provinces	Sickness or injury in the past four weeks; Self-reported health	Fixed-effect models, IV¶,DD§ estimation,PSM†	Overall health status showed no improvement.	**Sickness or injury in the past four weeks:**2.8% decrease (*P*<0.05).**Self-reported health** ‡ **:**No significant effect.	Yes: 9No: 0Unclear: 1
Lipow (2010)[28]	20002004	O×OO×ORepeated cross-sectional design	CHNSHeilongjiang, Shandong, and Hunan province	660 individuals	Sickness or injury in the past four weeks	Non-pooled and pooled OLS and linear probability models	Participating in the NCMS reduced the probability of being sick or injured in the past four weeks.	**Sickness or injury in the past four weeks:**18.81% decrease (*P*<0.05).	Yes: 9No: 0Unclear: 1
Yu et al. (2010)[38]	2006	O×OPosttest-only design with control group	Ningxia and Shandong province.	6,147 households and 22,636 individuals (20889 NCMS and 1739 non-NCMS members)	Sickness or injury in the past four weeks	Chi-square test	No significant differences of perceived health status between the two groups.	**Sickness or injury in the past four weeks:**NCMS members: 24.3%Non-NCMS members: 26.2% (*P* = 0.082)	Yes: 8No: 1Unclear: 0
Chu (2010)[25]	200020042006	O×OO×OPre-post design with control group	CHNSLiaoning, Heilongjiang, Jiangsu, Shandong, Henan, Hubei, Hunan, Guangxi, and Guizhou province	10,443 individuals (3481 for each round)	Sickness or injury in the past four weeks; Self-reported health	Single difference, double difference, and triple difference methods	No significant effect was found between NCMS members and internal controls.Sickness or injury in the past four weeks was decreased among NCMS members compared with external controls.	**Sickness or injury in the past four weeks:**Internal control: RD** = −0.054External control:RD = −0.090 (*P*<0.01)**Self-reported health:**Internal control: RD = −0.004External control: RD = 0.064	Yes: 9No: 0Unclear: 1
Feng (2009)[26]	2007	O×OPosttest-only design with control group	Hubei and Sichuan province	6003 households and 24,717 individuals (2969 NCMS members and 3034 non-NCMS members)	Sickness or injury in the past two weeks	Probit model	Participating in the NCMS reduced the probability of being sick or injured in the past two weeks.	**Sickness or injury in the past two weeks:**RD = −0.053(*P* = 0.0000)	Yes: 6No: 3Unclear: 0
Li et al. (2008)[27]	2005	O×OPosttest-only design with control group	Heilongjiang province	607 households and 607 individuals (306 NCMS and 301 non-NCMS members)	Sickness or injury in the past two weeks	Chi-square test	No significant differences of Sickness or injury in the past two weeks between the two groups.	**Sickness or injury in the past two weeks:**NCMS members: 11.52%Non-NCMS members: 11.05% (*P* = 0.170)	Yes: 6No: 3Unclear: 0
Liu (2006)[29]	20032005	O×OORepeated cross-sectional design	Henan province	2003:37585 individuals (9720 households) in 18 Non-NCMS counties2005:34492 individuals (9180 households) in 17 NCMS counties and 58861 individuals (14440 households) in 8 non-NCMS counties	Sickness or injury in the past two weeks	Chi-square test	Sickness or injury in the past two weeks among the NCMS members was lower than that of non-NCMS members.	**Sickness or injury in the past two weeks:**Pre-NCMS: 10.6%Post-NCMS: 5.7% (*P* = 0.000)Control: 9.1% (*P* = 0.000)	Yes: 6No: 3Unclear: 0
Meng (2009)[30]	20002006	O×OO×OPre-post design with control group	CHNSJiangsu province	364 individuals(NCMS members: 288 individuals; non-NCMS members: 76 individuals)	Sickness or injury in the past four weeks	NA	No effect of NCMS on sickness or injury in the past four weeks.	**Sickness or injury in the past four weeks:**NCMS members:2000∶9.02%; 2006∶18.75%Non-NCMS members:2000∶2.63%; 2006∶9.21%(Author: DD method, *P* = 0.5324)	Yes: 5No: 4Unclear: 1
Miao & Zhang (2008)[31]	2006	O×OPosttest-only design with control group	Jiangsu, Shandong, Henan, and Sichuan province	1046 individuals (1354 households)	Self-reported health	Z test	No significant differences of perceived health status between NCMS members and controls.	**Self-reported health:**NCMS members vs. external controls: 36.11% vs. 40.71%NCMS members vs. internal controls: 54.03% vs. 43.59%	Yes: 4No: 4Unclear: 1
Shen & Jiang (2008)[32]	2006	O×OPosttest-only design with control group	Shaanxi and Sichuan province	2273 individuals (550 households)	Sickness or injury in the past four weeks	Logistic regression	Participating in the NCMS increased the probability of being sick or injured in the past four weeks.	**Sickness or injury in the past four weeks:**OR = 1.483 (*P*<0.001)	Yes: 5No: 3Unclear: 1
Center for Health Statistics and Information, Ministry of Health, China (2007)[33]	2005	O×OPosttest-only design with control group	17 provinces: Shanxi, Inner Mongolia, Jilin, Heilongjiang, Shanghai, Zhejiang, Anhui, Shandong, Henan, Hubei, Guangdong, Guangxi, Chongqing, Yunnan, Shaanxi, Gansu, and Qinghai province	54760 individuals in 27 NCMS counties	Sickness or injury in the past two weeks; Self-reported health	NA	Overall health status among NCMS members became worse compared with non-NCMS members.	**Sickness or injury in the past two weeks:**NCMS members: 20.3%Non-NCMS members: 17.6%(Author: χ^2^ test, *P* = 0.0000)**Self-reported health:**NCMS members: 73.2%Non-NCMS members: 74.7%(Author: χ^2^ test, *P*<0.001)	Yes: 5No: 4Unclear: 0
Wu & Shen (2010)[36]	20042006	OOO×OPre-post design with control group	CHNSHenan, Hubei, Heilongjiang, Liaoning, and Guizhou province	NCMS: 991 individuals (532 households)Non-NCMS: 1587 individuals (842 households)	Self-reported health	PSM estimation,DD method	Self-reported health showed limited improvement.	**Self-reported health:**NCMS members:2004∶58.95%; 2006∶61.25%Non-NCMS members:2004∶59.98%; 2006∶59.36%RD = 2. 75% (*P*<0.1)	Yes: 8No: 0Unclear: 2

O indicates survey and X indicates implementation of NCMS in “Study design” column.

* CHNS: China Health and Nutrition Survey.

¶ IV: Instrumental variable estimation.

§ DD: Difference-in-differences estimation.

† PSM: Propensity score matching.

‡ Self-reported health: The percentage of “very good” or “good”.

** RD: Difference between NCMS members and non-NCMS members.

Of the six studies examining the effect of NCMS on sickness or injury in the past four weeks, Lei and Lin [8], Chu [25], and Lipow [28] utilized regression models to analyze panel data (with pre-post design with control group or repeated cross-sectional design). They found that NCMS reduced sickness or injury in the past four weeks by 2.8% (SE: 1.4%) (PSM, DD), 9.0% (SE: 3.1%) (DD) and 18.81% (SE: 7.75%) (OLS), respectively. Another study with panel design showed no effect without controlling any confounding factors [30]. The results from two cross-sectional studies are mixed: Shen and Jiang found that enrollment in NCMS significantly increased the probability of being sick or injured in the past four weeks (OR = 1.483, *P*<0.001) [32], and Yu et al. suggested no significant differences between the NCMS and non-NCMS [38].

Of the four studies examining the effect of NCMS on sickness or injury in the past two weeks, Feng used a probit model to analyze cross-sectional data and estimated that NCMS reduced sickness or injury in the past two weeks by 5.3% [26], which is consistent with the panel study by Liu [29]. Another two cross-sectional studies utilized chi-square tests without controlling any confounding factors and showed conflicting results: Li et al. suggested no significant differences between the NCMS and non-NCMS [27]; and Liu’s study found that participating in NCMS significant increased the probability of being sick or injured in the past two weeks [33].

Of the five studies examining the effect of NCMS on self-reported health, scaled as “very good”, “good”, “fair”, “poor” and “very poor”, three panel studies used the DD method and/or PSM estimation and showed no improvement or very limited improvement (*P*<0.1) of self-reported health after NCMS [8], [25], [36]. In addition, two cross-sectional studies without controlling any confounding factors showed no effect or worse health outcome (73.2% of the NCMS members and 74.7% of controls were assessed “very good” or “good” by themselves) [31], [33].

### 4. NCMS and Household Catastrophic Health Expenditure

Table 2 shows four studies measuring household catastrophic health expenditure, which set 40% or 50% of household income as the catastrophic threshold proportion. Two panel studies by Wagstaff et al. [35] and MoH [33] used PSM estimation and/or the DD method and suggested no effect. Another panel study reported that NCMS reduced the incidence of catastrophic health expenditure [37]. In their study, the incidences of catastrophic health expenditure, defined as 50% or more of net income before and after NCMS, were 11.36% and 6.08%, respectively, and the chi-square test showed the differences in these percentages to be statistically significant. Sun utilized cross-sectional design and indicated that 6.23% of NCMS members faced catastrophic health spending, which was lower than 13.10% for non-NCMS members. No statistical analysis was performed on these differences [34]. In addition, Wagstaff et al. also set 10% or 20% of household income as the criteria for catastrophic health spending, and found that NCMS increased the incidence of catastrophic household out-of-pocket payments [35].

**Table 2:** Characteristics of quasi-experimental studies estimating effects of NCMS on catastrophic health expenditure.

Author	Year data collected	Study design	Sample site and data source	sample size	Measures	Statistic analysis	Findings	Outcomes	Study quality
Wagstaff et al. (2007)[35]	20032005	O X OO X ORepeated cross-sectional design	2003: National HealthService Survey (NHSS)12 provinces:Chongqing, Guangdong, Heilongjiang, Inner Mongolia, Qinghai Shandong, Shanxi, Shanghai, Gansu, Zhejiang, Henan, and Guangxi province	8,476 households inthe 15 counties(5641 householdsin 10 NCMScounties)	Household annualhealth spendingdivided by householdincome: >10%, 20%, 40%	PSM*estimation,DD¶method	NCMS appeared tohave increased theincidence ofcatastrophic householdout-of-pocketpayments, at least wherethe catastrophicthreshold is 20% orless of income.	**>10% of income:** coeff = 0.028 t = 2.34**>20% of income:** Coeff = 0.039 t = 4.09**>40% of income:** Coeff = 0.007 t = 1.19	Yes: 8No: 0Unclear: 1
Sun (2005)[34]	2004	OX OPosttest-only design with control group	Shanxi province	612 households	Household annualhealth spendingdivided by householdnet income: > = 40%	NA	NCMS reduced the incidence of catastrophic health expenditure.	**> = 40% of net income:**NCMS members: 6.23%Non-NCMS members: 13.10%	Yes: 5No: 3Unclear: 0
Yan et al.(2009)[37]	20032007	O X ORepeated cross-sectional design	Shaanxi province	2003∶132 households2007∶540 households	Household annualhealth spendingdivided by householdnet income: > = 50%	NA	NCMS reduced the incidence of catastrophic health expenditure.	**> = 50% of net income:**2003∶11.36%2007∶6.08%(Author: χ^2^ test, *P*<0.05)	Yes: 3No: 5Unclear: 0
Center for Health Statistics and Information, Ministry of Health, China (2007)[33]	20032005	O X OO X ORepeated cross-sectional design	17 provinces: Shanxi, Inner Mongolia, Jilin, Heilongjiang, Shanghai, Zhejiang, Anhui, Shandong, Henan, Hubei, Guangdong, Guangxi, Chongqing, Yunnan,Shaanxi, Gansu, andQinghai province	10 NCMS counties:2003(pre-NCMS):18473(5815 households)2005(post-NCMS):19005(5830 households)5 Non-NCMS counties:2003∶11158(2949 households)2005∶11109(2946 households)	Household annualhealth spendingdivided by householdincome: >40%	PSM estimation	NCMS showed no improvement.	**>40% of income:** NCMS members: 6.35%Non-NCMS members: 7.49% (*P*>0.05)	Yes: 6No: 2Unclear: 0

O indicates survey and X indicates implementation of NCMS in “Study design” column.

* PSM: Propensity score matching.

¶ DD: Difference-in-differences estimation.

## Discussion

Although there is a growing body of literature regarding the effect of NCMS on health outcomes and/or alleviating catastrophic health expenditure, in this systematic review only fifteen studies could meet our inclusion criteria. The variation and methodological limitations among eligible studies (prior to Dec. 31, 2010) indicate that there is no sufficient and clear evidence to support the hypothesis that NCMS improves the health outcomes of rural population and/or decreases the catastrophic health expenditure of rural households.

Hadley [39], and Levy and Meltzer [20] reviewed the studies carried out in high-income countries, and summarized that health insurance improved health status to a certain extent. However, methodological limitations hampered the task of assessing causality. In 1999, Wagstaff and Yu examined the effects of the World Bank Health VIII project in Gansu province located in the northwest of China, which aimed to reestablish CMS, provide financial aid to the poor, and target the supply side, such as improving the infrastructure of township health centers. They found that self-reported health was not improved by the project [40]. In one of China’s western provinces, Wang et al. evaluated the impact of a community-based health insurance scheme conducted from 2003 to 2006 and suggested that it had a positive effect on the health status of enrollees [41]. The main differences between the scheme designed by Wang et al. [41] and NCMS lie in: (1) Their benefit packages are different - NCMS in the initial several years did not cover outpatient services in many counties. Patients might delay their health care service utilization until they were very sick and eligible for hospitalization service, which neither reduce financial burden for the enrolled nor improve their health status. (2) The provider payment methods are different – pay by salary plus performance-based bonus (Wang et al. [41]) versus fee-for-service (NCMS). Yip and Hsiao’s study using the same data with Wang et al. confirmed the above point [42]. In addition, Wagstaff et al. found that poor residents participating in NCMS still had financial barriers to access health services due to high co-payments [35].

On the other hand, one determinant for catastrophic health expenditure identified by Xu et al. [43] was a lack of health insurance. However, Wagstaff and Lindelow analyzed three household surveys in China from 1991 to 2003. They suggested that health insurance (CMS, Urban Employee Basic Medical Insurance, private insurance and so on) increased the risk of high and catastrophic spending due to insurance encouraging people to seek care when sick and to seek care from higher-level providers [44]. Similar to NCMS, although the Ministry of Health revealed the overall real hospitalization reimbursement was 41.5% nationally in 2009 [4], expanding coverage of NCMS did not automatically improve financial protection in this systematic review. Consistent with the review by You and Kobayashi, they found that NCMS had no statistically significant effect on average out-of-pocket medical expenses or on reducing the risk catastrophic expenses [45], which may be due to the increase of the availability of health services or higher-level health services to raise the proportion of households facing catastrophic health expenditure [43]–[44].

For health intervention strategies, as Peters et al. stated, some of the success factors, such as strong leadership, good management systems, clear accountabilities, and incentives to support performance, could lead to improve health service delivery in low- and middle-income countries [46]. As for ineffectiveness of NCMS, other reasons may be attributed to limited financing, inadequate management capacity, the different NCMS programs designed and implemented by the local governments, and varied methodological quality of studies. The year data were collected in the included studies is from 2003 to 2007. Starting in 2009, there has been a significant increase in reimbursement. The government increased its contribution in 2009–2011. 850 billion RMB (1 USD ≈ 6.81 RMB, Jan. 2009) was committed from central and local governments over these three years to support the healthcare reform. For NCMS it meant an increase in premium subsidies from 80 RMB in 2009, 120 RMB in 2010, to 200 RMB in 2011 [47], which may meet the reimbursement level for catastrophic health expenses. Because of the price elasticity of health care expenditures [48], some researchers continue to argue that increased funding is still insufficient to meet the promised level of reimbursement for catastrophic expenses.

Several factors complicate the findings of this systematic review. Firstly, health outcomes measurement played a critical role in the relationship between NCMS and health outcomes. Health outcomes in the current study include prevalence of sickness or injury in the last two or four weeks, proportion of self-reported health, and mortality. Due to the moral hazard problem, the insured are inclined to be diagnosed with more conditions and use more treatment, while the uninsured may be more likely to die without treatment. In this case, mortality measured as a health outcome could reflect the real effects of NCMS. However, no eligible studies in this systematic review (till December 31, 2010) measured mortality as a health outcome. According to a very recent paper by Chen and Jin [49], the raw numbers showed a lower mortality of children under 5 years old and pregnant women after NCMS was introduced. However, the effect went away after controlling for endogenous participation, which is consistent with our findings. In addition, self-reported health is regarded as a strong predictor of mortality demonstrated by varied studies [50], [51], and findings from the five studies with self-reported health are homogeneous in this study.

Secondly, selection from voluntary NCMS participation leads to biased comparison groups among cross-sectional studies, and endogeneity of NCMS resulted in underestimation or overestimation of its effect on health outcome. Therefore, main conclusion was supported by the findings of panel studies with regression analysis controlling selection bias and endogeneity in this systematic review.

At the same time, several limitations need to be borne in mind in interpreting our findings. Firstly, the number of eligible studies was only fifteen although there is a growing body of literature on NCMS. Moreover, as a social experiment in universal insurance coverage, there is increasingly less chance of finding a non-NCMS control group, which means that very few relevant studies could be considered in a future update of this systematic review. Secondly, although the included studies cover most of mainland China, methodological limitations, such as the lack of controlling confounders, constrain making generalizations across the rural population. This is consistent with the argument many researchers make–health insurance is an endogenous variable, and studies vary in the degree they address this endogeneity problem [20]. Thirdly, under the two principles of voluntary participation and catastrophic illnesses coverage [52], NCMS operates at the county level, and varies in design and implementation across counties. This results in different models of NCMS in China based on the reimbursement of inpatient expenses, combined with household savings account for general outpatient expenses, with large outpatient expenses due to selected chronic diseases, and with pooling revenue for general outpatient expenses [4]. However, only three studies mentioned the reimbursement models [30], [33], [35], and NCMS was regard as dichotomous variable in all of the included studies.

The NCMS revenue sharply increased annually from 3.0 billion RMB in 2004 to 130.8 billion RMB in 2010 [4], [53]. NCMS is a highly subsidized health insurance scheme, and government financing comprised more than half of the annual NCMS pooling revenue. For example, central and local government financing in 2009 accounted for 28.55% and 49.98% of NCMS revenue, respectively [4]. It is crucial to transform this given amount of spending on health into effective and efficient health services so as to meet the two universal rural health insurance goals of helping rural residents minimize catastrophic financial risks and improve their health status.

Firstly, payment method reform is an alternative to control unnecessary medical cost and improve the health service quality. In the recent decades Chinese doctors earned money on a fee-for-service basis, and it is essential to alter their incentives in order to make NCMS reach its goals. Pilot reform for the provider payment method has been tried in some areas. For example, in Lufeng county of Yunnan province they changed from fee-for-service to per bed-diem in county hospitals and township health centers. This resulted in a positive change in health provider behaviors and reduced out-of-pocket payments for patients [54].

Secondly, NCMS benefit package should fit into the disease profile and health expenditure pattern of the population [42]. Chronic diseases, needing not only hospitalization service but also ambulatory care and drugs, are responsible for 68.8% of the total disease burden in China. Moreover, its epidemic, which has become the main cause of catastrophic health expenditure, will rise in the future [55]. At the same time, in some counties where NCMS reimburses inpatient expenses combined with household savings accounts, NCMS does not address large outpatient expenses due to chronic conditions. Therefore, as Bloom stated, it is important to increase the willingness of individuals to shift their contribution from household saving accounts to a NCMS risk pool so as to improve financial protection [56]. In addition, preventive health services for chronic conditions such as disease screening and rehabilitation services could be considered to be included in the benefit package. In the latest notice issued by Ministry of Health, the rehabilitation service will be included in NCMS reimbursement [47].

Lastly, information systems including electronic medical records in health facilities and its interlinkage across health facilities are essential to real-time expense reimbursement, further reform fee-for-service payment method to comprehensive but effective payment methods such as diagnosis-related groups (DRGs) payment.

In spite of methodological limitations of individual studies included, our study still provides evidence-based assessment on the effect of NCMS. The construction of an effective NCMS needs its own evolved design. China is developing a more comprehensive health system, and its effort in the rural health system is not isolated. Improvements in the health care of its rural population plays a significant role in its effort to construct a “harmonious society” [57].

## Supporting Information

Checklist S1 PRISMA Flowchart Checklist.
(DOC)

Table S1 Database for literature search.
(DOC)

Appendix S1 Detailed search strategy.
(DOC)

Appendix S2 Inclusion form level 1 screening.
(DOC)

Appendix S3 Inclusion form level 2 screening.
(DOC)

Appendix S4 Quality assessment criteria for quasi-experiment study.
(DOC)
