# Supplementary material for: The Effect of New Cooperative Medical Scheme on Health Outcomes and Alleviating Catastrophic Health Expenditure in China: A Systematic Review
Source: PLoS One. 2012 Aug 20;7(8):e40850. doi: 10.1371/journal.pone.0040850 (PMC3423411; doi:10.1371/journal.pone.0040850)
Supplement: Table S1 — Database for literature search. (DOC) [file pone.0040850.s002.doc]

**Appendix 5: Results of literature search**

Table 1. Database for literature search

| Database | Language | Literature type | Hits |
| --- | --- | --- | --- |
| CNKI (China National Knowledge Infrastructure) | Chinese | Academic paper | 3344 |
|  |  | Conference paper | 189 |
|  |  | Dissertation(PhD) | 89 |
|  |  | Dissertation(Master) | 485 |
| Chongqing VIP database (a full text issues database of China) | Chinese | Academic paper | 1329 |
| CMCI(China Biomedicine Database) | Chinese | Academic paper | 598 |
| PubMed | English | Academic paper | 24 |
| ISI Web of Science [with Conference Proceedings](http://metasearch.kib.ki.se/databases/proxy/LIB45278) | English | Academic paper | 48 |
|  |  | Conference paper | 9 |
| ProQuest Digital Dissertations | English | Dissertation | 6 |
| Other sources | Chinese/English | Book, Working paper | 2 |
| Total |  |  | 6123 |
| Total after duplicates removed |  |  | 4699 |

Table 2. Relevant literature

| Literature type | Language | Hits |
| --- | --- | --- |
| Academic paper | Chinese | 3865 |
|  | English | 54 |
| Dissertation | Chinese | 574 |
|  | English | 6 |
| Conference paper | Chinese | 189 |
|  | English | 9 |
| Working paper | Chinese | 0 |
|  | English | 1 |
| Book | Chinese | 1 |
|  | English | 0 |
| Total | Chinese | 4629 |
|  | English | 70 |
