# Supplementary material for: The Effect of New Cooperative Medical Scheme on Health Outcomes and Alleviating Catastrophic Health Expenditure in China: A Systematic Review
Source: PLoS One. 2012 Aug 20;7(8):e40850. doi: 10.1371/journal.pone.0040850 (PMC3423411; doi:10.1371/journal.pone.0040850)
Supplement: Appendix S1 — Detailed search strategy. (DOC) [file pone.0040850.s003.doc]

**Appendix 1: Detailed search strategy**

**CNKI** (http://dlib.edu.cnki.net/kns50/)

(1) Chinese Journal full-text database (CJFD)

2003-2010

#1 New Cooperative Medical Scheme[Title]

#2 New Cooperative Medical Scheme [Keywords]

#3 #1 OR #2

#4 Health[Subject headings]

#5 Cost[Subject headings]

#6 Expenditure[Subject headings]

#7 Reducing the impoverishing effect of healthcare payments [Subject headings]

#8 OR (#4 - #7)

#9 #3 AND #8

(2) China Proceedings of Conference Full-text Database (CPCD)

(3) China Doctoral Dissertations Full-text Database (CDFD)

(4) Master Theses Full-text Database

2003-2010

#1 Health[Subject headings]

#2 Cost[Subject headings]

#3 Expenditure[Subject headings]

#4 Reducing the impoverishing effect of healthcare payments [Subject headings]

#5 New Cooperative Medical Scheme [Subject headings]

#6 #1 OR #2 OR #3 OR #4 AND #5

**Chongqing VIP database** (http://vip.calis.edu.cn/)

2003-2010

#1 Health[All fields]

#2 Cost[All fields]

#3 Expenditure[All fields]

#4 Reducing the impoverishing effect of healthcare payments [All fields]

#5 New Cooperative Medical Scheme [Title/Keywords]

#6 #1 OR #2 OR #3 OR #4 AND #5

**CMCI** (http://202.204.190.39:1015/cdweb/leadpage.htm)

2003-2010

#1 Health[All fields]

#2 Cost[All fields]

#3 Expenditure[All fields]

#4 Reducing the impoverishing effect of healthcare payments [All fields]

#5 New Cooperative Medical Scheme [All fields]

#6 #1 OR #2 OR #3 OR #4 AND #5

**PubMed** (http://www.ncbi.nlm.nih.gov/sites/entrez?db=pubmed&itool=toolbar)

2003-2010

#1 “New Cooperative Medical Scheme” [TW]

#2 “New Cooperative Medical System” [TW]

#3 “New Rural Cooperative Medical Scheme” [TW]

#4 “New Rural Cooperative Medical System” [TW]

#5 OR (#1 - #4)

#6 “health” [MESH]

#7 “payment” [TW]

#8 “cost” [TW]

#9 “expenditure”[TW]

#10 “catastrophic”[TW]

#11 OR (#6 - #10)

#12 #5 AND #11

**ISI Web of Science** [**with Conference Proceedings**](http://metasearch.kib.ki.se/databases/proxy/LIB45278) (http://apps.webofknowledge.com/WOS_GeneralSearch_input.do?product=WOS&search_mode=GeneralSearch&SID=1E8g8Cbe2LD3I9@EBjA&preferencesSaved=)

2003-2010

#1 “New Cooperative Medical Scheme” [TS]

#2 “New Cooperative Medical System” [TS]

#3 “New Rural Cooperative Medical Scheme” [TS]

#4 “New Rural Cooperative Medical System” [TS]

#5 OR (#1 - #4)

#6 “health” [TS]

#7 “payment” [TS]

#8 “cost” [TS]

#9 “expenditure” [TS]

#10 “catastrophic” [TS]

#11 OR (#6 - #10)

#12 #5 AND #11

**ProQuest** (http://search.proquest.com/index)

Dissertations & Theses

2003.1.1-2010.12.31

#1 “New Cooperative Medical Scheme” [Citation and abstract]

#2 “New Cooperative Medical System” [Citation and abstract]

#3 “New Rural Cooperative Medical Scheme” [Citation and abstract]

#4 “New Rural Cooperative Medical System” [Citation and abstract]

#5 OR (#1 - #4)

#6 #5 AND “health”[Citation and abstract]

#7 #5 AND “cost”[Citation and abstract]

#8 #5 AND “payment”[Citation and abstract]

#9 #5 AND “catastrophic”[Citation and abstract]

#10 #5 AND “expenditure”[Citation and abstract]

#11 OR (#6 - #10)
