# Supplementary material for: The Effect of New Cooperative Medical Scheme on Health Outcomes and Alleviating Catastrophic Health Expenditure in China: A Systematic Review
Source: PLoS One. 2012 Aug 20;7(8):e40850. doi: 10.1371/journal.pone.0040850 (PMC3423411; doi:10.1371/journal.pone.0040850)
Supplement: Appendix S2 — Inclusion form level 1 screening. (DOC) [file pone.0040850.s004.doc]

**Appendix 2: Inclusion form level 1 screening**

**Questions for promoting / excluding records from title/abstract reading**

**Ref number: _____________ First author, year_________________________**

**Reviewer decision (after completing the form):**

Promote  (All questions are answered "YES" or "Can't tell")

Exclude  (Some questions are answered "No")

**Final decision (after discussion):**

Promote  (All questions are answered "YES" or "Can't tell")

Exclude  (Some questions are answered "No")

**1. Are there reported data?**

Yes  No  Unclear

**2. Is it a study pertaining to NCMS?**

Yes  No  Unclear

**3. Are the reported data either regarding the effect of NCMS on health outcome and/or alleviating catastrophic expenditure?**

Yes  No  Unclear
