# Supplementary material for: The Effect of New Cooperative Medical Scheme on Health Outcomes and Alleviating Catastrophic Health Expenditure in China: A Systematic Review
Source: PLoS One. 2012 Aug 20;7(8):e40850. doi: 10.1371/journal.pone.0040850 (PMC3423411; doi:10.1371/journal.pone.0040850)
Supplement: Appendix S3 — Inclusion form level 2 screening. (DOC) [file pone.0040850.s005.doc]

**Appendix 3: Inclusion form level 2 screening**

**Questions for including / excluding records from full text reading**

**REVIEW 1: "Causal effects of NCMS on Health outcome and/or alleviating the catastrophic expenditure "**

**Ref number: _____________ First author, year_________________________**

**Reviewer decision (after completing the form):**

Include  (All questions are answered "YES")

Discuss  (Some questions are answered “CAN’T TELL”)

Exclude  (Some questions are "NO")

**Final decision (after discussion):**

Include  (All questions are answered "Yes")

Exclude  (Some questions are answered "No")  Reason /#: __

**1. Are there reported data from a primary study with the following design? Design = Quasi-experiment**

Yes  No  Unclear

**2. Are the study participants members of NCMS?**

Yes  No  Unclear

**3. Is NCMS group compared with non-NCMS group?**

Yes  No  Unclear

**4. Is an index of health outcome/alleviating the catastrophic expenditure an outcome?**

Yes  No  Unclear

**Comments: ______________________________________________________**

**_______________________________________________________________**
