# Supplementary material for: The Effect of New Cooperative Medical Scheme on Health Outcomes and Alleviating Catastrophic Health Expenditure in China: A Systematic Review
Source: PLoS One. 2012 Aug 20;7(8):e40850. doi: 10.1371/journal.pone.0040850 (PMC3423411; doi:10.1371/journal.pone.0040850)
Supplement: Appendix S4 — Quality assessment criteria for quasi-experiment study. (DOC) [file pone.0040850.s006.doc]

**Appendix 4: Quality assessment criteria for quasi-experiment study**

1. Were appropriate procedures used to increase the likelihood that relevant characteristics of participants in the sample were comparable between NCMS and non-NCMS?
2. Was there adequate description of how NCMS was implemented in the study area?
3. Was the period between the implementation of NCMS and outcome more than 1 year (health outcomes)?
4. Was measurement method same for NCMS and non-NCMS?
5. Was data available on attrition rates among NCMS and non-NCMS samples for longitudinal cohort study?
6. Did the research report control relevant confounders between groups prior to the intervention (either in the design (e.g. stratification, matching) or analysis)?
7. Did the research report include inferential statistics (*P* value)?
8. Did the research report include effect size calculations (confidence interval)?
9. Did the research report include discussion of possible biases and caveats (or limitations)?
10. Did the research report include the generalizability of the study in the discussion?

Note: *Adapted from Loevinsohn (1990); Thomas et al. (2004); Gersten et al.(2005)
